# Serotonin activates glycolysis and mitochondria biogenesis in human breast cancer cells through activation of the Jak1/STAT3/ERK1/2 and adenylate cyclase/PKA, respectively

**DOI:** 10.1038/s41416-019-0640-1

**Published:** 2019-12-10

**Authors:** Mauro Sola-Penna, Larissa P. Paixão, Jessica R. Branco, Alan C. Ochioni, Jamille M. Albanese, Davi M. Mundim, Daniela Baptista-de-Souza, Claudia P. Figueiredo, Wagner S. Coelho, Mariah C. Marcondes, Patricia Zancan

**Affiliations:** 1 Laboratório de Enzimologia e Controle do Metabolismo, Departamento de Biotecnologia Farmacêutica, Faculdade de Farmácia, Universidade Federal do Rio de Janeiro, 21941-902 Rio de Janeiro, RJ Brazil; 2 Laboratório de Oncobiologia Molecular, Departamento de Biotecnologia Farmacêutica, Faculdade de Farmácia, Universidade Federal do Rio de Janeiro, 21941-902 Rio de Janeiro, RJ Brazil; 3 Departamento de Psicologia, Universidade Federal de São Carlos, 13565-905 São Carlos, SP Brazil; 4 Nucleo de Neurociências da Faculdade de Farmácia, Departamento de Biotecnologia Farmacêutica, Faculdade de Farmácia, Universidade Federal do Rio de Janeiro, 21941-902 Rio de Janeiro, RJ Brazil; 5 Universidade Estadual da Zona Oeste, 23070-200 Rio de Janeiro, RJ Brazil; 6 Instituto Nacional de Câncer José Alencar Gomes da Silva, 20230-130 Rio de Janeiro, RJ Brazil

**Keywords:** Breast cancer, Mechanisms of disease

## Abstract

**Background:**

Although produced by several types of tumours, the role of serotonin on cancer biology is yet to be understood.

**Methods:**

The effects of serotonin (5-HT) on human breast cancer cells proliferation, signalling pathways and metabolic profile were evaluated by cytometry, western blotting, qPCR, enzymology and confocal microscopy.

**Results:**

Our results revealed that incubation of MCF-7 cells with 10 µM 5-HT increased cell growth rate by 28%, an effect that was prevented by the 5-HTR_2A/C_ antagonist, ketanserin. Conversely, increasing concentrations of 5-HT promoted glucose consumption and lactate production by MCF-7 cells. We also showed that increased glucose metabolism is provoked by the upregulation of pyruvate kinase M2 (PKM2) isoform through 5-HTR_2A/C_-triggered activation of Jak1/STAT3 and ERK1/2 subcellular pathways. However, we noticed a decrease in the rate of produced lactate per consumed glucose as a function of the hormone concentration, suggesting a disruption of the Warburg effect. The latter effect is due to 5-HTR_2A/C_-dependent mitochondrial biogenesis and metabolism, which is triggered by adenylyl cyclase/PKA, enhancing the oxidation of lactate within these cells.

**Conclusions:**

We showed that serotonin, through 5-HTR_2A/C_, interferes with breast cancer cells proliferation and metabolism by triggering two distinct signalling pathways: Jak1/STAT3 that boosts glycolysis through upregulation of PKM2, and adenylyl cyclase/PKA that enhances mitochondrial biogenesis.

## Background

In addition to its well-known action as a neurotransmitter, serotonin (5-HT) is an endocrine hormone involved in various pathophysiological processes.^1,2^ In the gut, for instance, where this hormone is primarily synthesised by the enterochromaffin cells, 5-HT controls gastrointestinal motility, secretions, inflammation and epithelial cell development among other functions.^3^ The hormone is also clinically involved in liver regeneration after partial hepatectomy, where it plays a key role in inducing hepatocyte mitosis.^4^ This mitogenic activity of 5-HT is relevant for the growth and differentiation of diverse types of cancer, which explains the association between the levels of this hormone/neurotransmitter and tumour aggressiveness and prognosis among those tumours, including breast cancer.^5–7^ In this context, 5-HT has been also linked to the development of different cancers, including hepatocellular carcinoma, colon and breast cancers.^4,6–8^ In breast cancer specifically, 5-HT plays a crucial role in tumour development, via stimulating proliferation of breast cancer cells and prompting inappropriate cell survival microenvironmental characteristics.^8^ Moreover, since breast cancer cells produce high levels of 5-HT, free 5-HT plasma levels serve as early detection markers for breast cancer.^5^ Nevertheless, the exact role of 5-HT in breast cancer cell biology is yet to be understood, and the mechanisms by which 5-HT promotes its effects on carcinogenesis have not been deciphered so far. However, in non-tumoural tissues, serotonin activates glycolysis,^9–11^ which is considered the major pathway supporting cancer cells metabolism and division.^12^

It has been hypothesised that cancer cells adapt their metabolic profile to a unique pattern named ‘Warburg effect'. This effect is instigated by the tendency of cancerous cells to consume high amounts of glucose through the glycolytic flux—primarily fermenting glucose into two molecules of lactate—regardless of the oxygen supply to these cells even in aerobic conditions.^13^ This phenomenon, also called aerobic glycolysis, is considered a metabolic advantage to cancer cells, by providing a rapid and efficient source of energy and rendering it a potential therapeutic target in cancer.^14–16^ Indeed, aerobic glycolysis within cells has been shown to positively correlate with cancer aggressiveness and drug resistance.^17,18^ But recent findings have questioned the dependency of cancer cells on aerobic glycolysis, stating that cancer cell survival necessitates shifting to oxidative metabolism, the latter that is correlated to epithelial-to-mesenchymal transition, cancer invasion and aggressiveness.^19,20^

We have previously demonstrated that 5-HT triggers glycolysis in diverse tissues by activating glycolytic enzymes, which involves 5-HT_2A_ receptor activation.^9–11^ Since breast tumour cells produce and secrete 5-HT, we hypothesised that the hormone might have an autocrine action on these cells by modulating their energy metabolism. In the current work, we used the human breast cancer cell line MCF-7 to evaluate 5-HT effects on cancer metabolism. We compared the results with other cell lines, the non-tumorigenic MCF10A and the potentially metastatic MDA-mb-231. We showed that 5-HT modulates breast cancer biology through interfering with mitochondria biogenesis and shifting the fate of glycolysis products to oxidative metabolism, thus altering aerobic glycolysis and mitochondrial metabolism.

## Methods

### Materials

Human breast cancer cell lineages MCF-7 and MDA-mb-231 were, respectively, obtained from the Cell Bank of Rio de Janeiro (www.bcrj.org.br Duque de Caxias, RJ, Brazil) and gifted by Dr. Mitzi Brentani (USP, São Paulo, Brazil), and were grown and maintained at 37 °C (5% CO_2_ atmosphere) in Dulbecco’s Modified Eagle’s Medium (DMEM) with 25 mM glucose supplemented with 10% (vol/vol) heat-inactivated foetal bovine serum (FBS) and 5 mM L-glutamine (Invitrogen, São Paulo, SP, Brazil). The non-tumorigenic human breast cell line MCF10A was kindly supplied by Dr. M. Brentani (USP, São Paulo, Brazil), and was grown and maintained at 37 °C (5% CO_2_ atmosphere) in DMEM/F12 medium with 25 mM glucose supplemented with 10% (vol/vol) FBS, 0.02 mg/ml EGF, 5 mg/ml insulin, 1.25 mg/ml hydrocortisone, 0.1 mg/ml cholera toxin and 5 mM L-glutamine (Invitrogen, São Paulo, SP, Brazil). All the chemicals used were of the highest quality commercially available.

### Cell proliferation rate, apoptosis and viability

To evaluate the cell proliferation rate, cells were seeded in 24-well plates (4 × 10^4^ cells/well) and incubated for 3 h to adhere. After this short incubation, cells were treated with 5-HT (Sigma Chemicals Co., St. Louis, MO, USA) and/or ketanserin (Sigma Chemicals Co., St. Louis, MO, USA) and/or spiperone (Sigma Chemicals Co., St. Louis, MO, USA), accordingly, and then incubated for 18 h until reaching almost 50% confluency. Then, cells were treated with PE Annexin V reagent (1:20 dilution; BD Pharmigen, BD Biosciences, Franklin Lakes, NJ, USA) and 5 µM 7-AAD (BD Pharmingen, BD Biosciences, Franklin Lakes, NJ, USA), and the proliferation rate, apoptosis and cell viability were evaluated by counting the number of cells negative for both markers (viable), positive for Annexin V (apoptotic) and positive for 7-AAD staining (non-viable) by using a cell-sorting system (Muse^®^ Cell Analyzer, Merck Millipore, Billerica, MA, USA).

### Glucose consumption and lactate production

To evaluate glucose consumption and lactate production, cells were seeded in 96-well plates (8 × 10^3^ cells/well) and grown to 70% confluency. Then, the medium was replaced by a fresh one containing 2-(N-(7-nitrobenz-2-oxa-1,3-diazol-4-yl)amino)-2-deoxyglucose (2-NBDG; 1 mmol/mol glucose) to trace glucose consumption or not containing the tracer for lactate production. After 24-h incubation, the medium was removed to evaluate the amount of produced lactate, and cells were harvested and resuspended in phosphate-buffered saline (PBS; 137 mM NaCl, 2.7 mM KCl, 10 mM Na_2_HPO_4_ and 1.8 mM KH_2_PO_4_, pH 7.4) containing 0.1% bovine serum albumin (BSA, heat-shock fraction, fatty acid free, protease free, Sigma-Aldrich, St. Louis, MO, USA). Suspended cells were evaluated for glucose content by using a cell cytometer (Muse Cell Analyser, Merck Millipore, Billerica, MA, USA). A calibration curve was performed to determine the amount of glucose taken up by the cells. For determination of lactate production, the amount of lactate in the culture medium was evaluated by using a commercial kit (Labtest Diagnóstica S/A, Lagoa Santa, MG, Brazil). The lactate production/glucose consumption ratio was obtained by simply the product of these two measurements.

### Mitochondrial activity

Mitochondrial activity was assessed by the mitochondrial reductive potential via incubating the cells in the presence of 3-(4,5-dimethylthiazol-2-yl)-2,5-diphenyl tetrazolium bromide (MTT, Sigma Chemicals Co., St. Louis, MO, USA) and evaluating the formation of formazan products. Briefly, cells were seeded in 96-well plates (8 × 10^3^ cells/well) and grown to 70% confluency; then cells were treated with different concentrations of 5-HT for 24 h. After this period, the medium was removed and a solution of 5 mg/ml MTT was added followed by 3-h incubation at 37 °C. Then, the medium was removed, and 100 µl of DMSO was added to dissolve the formazan crystals formed, which was evaluated by measuring the absorbance at 560 nm subtracted by the absorbance at 670 nm.

### Enzymatic activities

The enzymatic activities were evaluated after the treatment for 24 h of cells seeded in 96-well plates and grown to 70% confluency. Following treatment, the medium was removed, and cells were lysed with 100 µl of 10 mM phosphate buffer (pH 7.4) by pipetting up and down several times. The cell-free homogenates were used for evaluating the enzymes’ activities. Hexokinase activity was assessed in a reaction medium containing 50 mM Tris-HCl (pH 7.4), 5 mM MgCl_2_, 1 mM glucose, 1 mM ATP, 150 mM KCl, 0.2 mM NADP^+^ and 0.2 mU/ml glucose-6-phosphate dehydrogenase. Phosphofructokinase activity was assessed in a reaction medium containing 50 mM Tris-HCl (pH 7.4), 1 mM MgCl_2_, 1 mM (NH_4_)_2_SO_4_, 1 mM fructose-6-phosphate, 0.1 mM ATP, 150 mM KCl, 0.2 mM NADH, 0.2 mU/ml aldolase, 0.4 mU/ml triosephosphate isomerase and 0.2 mU/ml α-glycerophosphate dehydrogenase. Pyruvate kinase activity was assessed in a reaction medium containing 50 mM Tris-HCl (pH 7.4), 1 mM MgCl_2_, 1 mM phosphor(enol)pyruvate, 1 mM ADP, 150 mM KCl, 0.2 mM NADH and 2 mU/ml lactate dehydrogenase. Lactate dehydrogenase activity: 50 mM Tris-HCl (pH 7.4), 1 mM MgCl_2_, 1 mM pyruvate and 0.2 mM NADH. Glucose-6-phosphate dehydrogenase activity was assessed in a reaction medium containing 50 mM Tris-HCl (pH 7.4), 5 mM MgCl_2_, 1 mM glucose-6-phosphate, 150 mM KCl and 0.2 mM NADP^+^. Reactions were started by adding 10 µl of the cell-free homogenates, and NAD(P) reduction/oxidation was followed spectrophotometrically at 340 nm. The slopes of the curves were used to determine the enzymatic activities. Succinate dehydrogenase was assessed in a reaction medium containing 50 mM Tris-HCl (pH 7.4), 5 mM MgCl_2_, 12 mM diethyl succinate, 0.2 mM 1-methoxy-5-methylphenazinium methyl sulfate and 1.2 mM nitro blue tetrazolium (NBT), in the absence and the presence of 12 mM malonate (a competitive inhibitor of succinate dehydrogenase). The reaction was started by adding 10 µl of cell-free homogenate, and the reduced NBT was evaluated spectrophotometrically at 600 nm. Succinate dehydrogenase activity was calculated by the slope of the curve.

### Quantitative PCR (qPCR)

For RNA extraction, cells were seeded in six-well plates (10^5^ cells/well) and grown to 70% confluency. Then the media were removed, and cells were treated according to the experiments. After the treatments, the media were removed, and 500 µl of Trizol reagent (ThermoFisher, Carlsbad, CA, USA) was added to each well, followed by homogenisation by up and down pipetting. After this, RNA extraction was performed by following the directions of the reagent. For cDNA synthesis, the high-capacity cDNA Reverse Transcription Kit (ThermoFisher, Carlsbad, CA, USA) was used. For qPCR, the GoTaq qPCR Master Mix (Promega, Fitchburg, WI, USA) was used and the reaction was performed in a QuantStudio 5 (ThermoFisher, Carlsbad, CA, USA). The programme for all amplifications was 2 min at 95 °C followed by 40 cycles of 15 s at 95 °C and 1 min at 60 °C. A dissociation curve was performed at the end of the experiment, and dissociation peak was analysed. The fold expression was calculated by the 2^–ΔΔCt^ method, as described previously.^21^ Expression levels are represented by the 2^–ΔCt^ method. *CCSER2* expression was used as reference gene (housekeeper), since its expression did not vary upon any of the used treatments (data not shown). Primers were designed by using Primer-blast tool,^22^ and all qPCR conditions were optimised by following international standards.^23^ The primers used are described in Table 1.

**Table 1:** Primers used for qPCR

Gene	Protein	Primer sequence		Amplicon length (bp)	Efficiency (%)
		Forward	Reverse		
*CCSER2*	Coiled-coil serine-rich protein 2	TGAGCAGAGGCTCTCCCTAT	GGAAAAAGGGCATCCCTCCA	72	99.5
*HK1*	Hexokinase 1	AGCACTGCTCCAGGTCCGGGCT	GCGGCCCTCCTGGACACCACCC	108	101.2
*HK2*	Hexokinase 2	GCCTGGCTAACTTCATGG	GAGCCCATGTCAATCTCC	361	104.2
*HTR1A*	5-hydroxytryptamine receptor 1A	CGAATCTTCGCGCTGCTTTT	TGATGTGGTGTTGTTGCCCT	97	97.3
*HTR1B*	5-hydroxytryptamine receptor 1B	CCCTGGAAAGTACTGCTGGT	TCCGGTACACTGTGGCAATC	91	96.8
*HTR1D*	5-hydroxytryptamine receptor 1D	CTCACCAGGAAGCTCCACAC	TGGAAACCAAGAGGTCGGTG	73	98.3
*HTR1E*	5-hydroxytryptamine receptor 1D	CGCACCGTCCACAAGGAAC	GTGGTCTCTTGTGTTCCATTTTCT	84	95.4
*HTR1F*	5-hydroxytryptamine receptor 1F	AGTGGGCCCTAGTGAAGGTAT	TCTGAGGTCAAGTTTTGATCAGATG	100	97.8
*HTR2A*	5-hydroxytryptamine receptor 2A	CCTGTATGGGTACCGGTGG	GATGGAGGCCGTGGAGAAG	85	101.1
*HTR2B*	5-hydroxytryptamine receptor 2B	TGGTGTGGTTAATTTCAATAGGCAT	TGGGTTGTCCACATCAGTCTC	74	99.3
*HTR2C*	5-hydroxytryptamine receptor 2C	TCCTTTATGATTATGTCTGGCCACT	GTGCATGATGGACGCTGTTG	92	95.9
*HTR3A*	5-hydroxytryptamine receptor 3A	TGCTCAGCCATGGGAAACC	TACATCTGTCCCTCGGGCTC	76	96.5
*HTR3B*	5-hydroxytryptamine receptor 3B	GGTAACAGAGTCCTCGCTGTATG	TGATAGATTGAAGCTGCGACCA	83	98.8
*HTR3C*	5-hydroxytryptamine receptor 3C	GGCCACCCCAAAGATGTCC	GCGCCTGATGGCCACATAA	70	102.1
*HTR3D*	5-hydroxytryptamine receptor 3D	CTCCCAGCCACTAGCACTTC	GCGAAGTAGACACCTCGCTT	83	100.8
*HTR3E*	5-hydroxytryptamine receptor 3E	GCAAGGCCACCGCAAAG	CTGATGGCCACATAGAACACGA	70	99.2
*HTR4*	5-hydroxytryptamine receptor 4	GACAGGCAGCTCAGGAAAATAAA	CAATGGCACCAAAGGGCATC	100	97.2
*HTR5A*	5-hydroxytryptamine receptor 5A	TCTACAAGGCTGCCAAGTTCC	GTCCTTCACCTCCACAGCTT	83	95.9
*HTR6*	5-hydroxytryptamine receptor 6	CATAGTCCAGGCCGTGTGC	ACAGTAACCCAGCCATGTGAG	70	99.7
*HTR7*	5-hydroxytryptamine receptor 7	GAGACCTGAGTTTGTGCTGAGG	ATACCGGTGGCCTCTTTTCTG	71	96.9
*LDHA*	Lactate dehydrogenase A	GGCACGTCAGCAAGAGGGAGA	GCAACTTGCAGTTCGGGCTGT	110	97.8
*LDHB*	Lactate dehydrogenase B	ACACCGCGTGATTGGAAGTGGA	TGGCAGCTGCTGGGATGAATGC	93	96.4
*PDK1*	Pyruvate dehydrogenase 1	TGCTTCGCGGAGCCGCCTTGG	CGCTCGGACGCCGGGCTGGA	103	95.0
*PFKM*	PFK isoform M	GGAGTGCGTGCAGGTGACCAAA	ATCACGGCCACTGTGTGCAACC	171	99.3
*PFKL*	PFK isoform L	CATGAATGCAGCTGTGCGCTCC	CCAGCCCACTTCTTGCACCTGA	118	102.6
*PFKP*	PFK isoform P	ACAGACACGTGCGACCGCAT	AGTGCACGACGTTGGACTGCA	187	98.5
*PKL*	PK L	TTGGGCGCTGCAACTTGGCGGG	CTGCCCTCGTTGGCCGGGGCT	90	97.7
*PKM1*	PK M1	GGCTGAGGACGTGGACCTCCGGG	AGCCAGGGCGCCATCCGGTCA	110	96.1
*PKM2*	PK M2	GCAAGGCATCTGATGTCC	CCCTTTGGCTGTTTCTCC	368	97.8
*SLC16A1*	MCT1	GTCATTGGAGGTCTTGGGCT	GCCAATGGTCGCCTCTTGTA	86	98.8
*SDHA*	SDH A	TGGGGAGTGCCGTGGTGTCA	TAGGTGCGCCCGTAGCCTCC	105	99.7
*SIRT3*	SIRT3	CCTGGAGGTGGAGCCTTTTG	AAGTCCCGGTTGATGAGCAG	78	101.1

All the primers were optimised for 600 nM use in the experiments

### Confocal microscopy

For confocal microscopy experiments, MCF-7 cells were seeded on coverslips placed in 24-well plates and incubated to reach 70% confluency. The medium was removed, and cells were treated in the presence or absence of 10 µM 5-HT or 10 µM 5-HT plus 0.1 µM ketanserin for 24 h at 37 °C. After treatment, 50 nM MitoTracker Green FM (Cat# M7514, Molecular Probes, ThermoFisher, Carlsbad, CA, USA) and 100 nM MitoTracker Red CMXRos (Cat# M7512, Molecular Probes, ThermoFisher, Carlsbad, CA, USA) were added to each well and incubated for 1 h at 37 °C. After incubation, the coverslips were used to evaluate fluorescent staining inside the cells by using an A1 confocal microscope (Nikon, Tokyo, Japan) at ×63 magnification. Three independent fields from each coverslip were used for analyses of one experiment. Each image acquired was a z-stack of 12–16 (0.33-μm depth) sections. Maximum projections of three consecutive optical sections (corresponding to 1-μm total depth) were generated from the middle of the original z-stack. The complete analysis was taken by using three independent experiments (*n* = 3). Quantification of the images was performed by using the software Image J64 (http://imagej.nih.gov/ij, NIH, USA).

### Mitochondrial evaluation by flow cytometry

Cells were seeded in 24-well plates (4 × 10^4^ cells/well) and incubated for 3 h to adhere. After this short incubation, cells were treated accordingly, and then incubated for 24 h until reaching almost 70% confluency. Then, cells were treated with 50 nM MitoTracker Green FM (Cat# M7514, Molecular Probes, ThermoFisher, Carlsbad, CA, USA) and 100 nM MitoTracker Red CMXRos (Cat# M7512, Molecular Probes, ThermoFisher, Carlsbad, CA, USA) for 1 h at 37 °C. Then, cells were detached and evaluated for the dye labelling by using a cell-sorting system (Muse^®^ Cell Analyzer, Merck Millipore, Billerica, MA, USA).

### Western blotting

For western blot, cells were seeded in six-well plates (10^5^ cells/well) and grown to 70% confluency. Then the media were removed, and cells were treated according to the experiments. After the treatments, the media were removed and a mild-RIPA buffer^24^ supplemented with protease inhibitor cocktail (Sigma-Aldrich, St. Louis, MO, USA) was added for total protein extraction. Protein extracts were submitted to SDS-PAGE (8% gels),^25^ followed by overnight transfer to nitrocellulose membranes at 30 V. Membranes were stained with Ponceau S, processed and de-stained by washing with distilled water. Then, the membranes were incubated overnight with the following antibodies: anti-5-HT2C receptor (dilution 1:1000, Cat# ab197776, Abcam, Cambridge, UK), anti-5-HT7 receptor (dilution 1:1000, Cat# ab61562, Abcam, Cambridge, UK), anti-β-actin (dilution 1:1000, Cat# 4967, Cell Signaling Technology, Danvers, MA, USA), anti-Akt (dilution 1:1000, Cat# 9272, Cell Signaling Technology, Danvers, MA, USA), anti-phospho-Akt (T308) (dilution 1:1000, Cat# 9275, Cell Signaling Technology, Danvers, MA, USA), anti-CREB (dilution 1:1000, Cat# 9197, Cell Signaling Technology, Danvers, MA, USA), anti-phospho-CREB (S133) (dilution 1:1000, Cat# 9196, Cell Signaling Technology, Danvers, MA, USA), anti-Hif-1α (dilution 1:1000, Cat# NB 100–449, Novus Biologicals, Centennial, CO, USA), anti-Jak1 (dilution 1:1000, Cat# 3332, Cell Signaling Technology, Danvers, MA, USA), anti-phospho-Jak1 (Y1034/1035) (dilution 1:1000, Cat# 3331, Cell Signaling Technology, Danvers, MA, USA), anti-PGC1α (dilution 1:1000, Cat# 2178, Cell Signaling Technology, Danvers, MA, USA), anti-PKM2 (dilution 1:1000, Cat# D78A4, Cell Signaling Technology, Danvers, MA, USA), anti-cPLA2 (dilution 1:1000, Cat# 2832, Cell Signaling Technology, Danvers, MA, USA), anti-phospho-cPLA2 (S505) (dilution 1:1000, Cat# 2831, Cell Signaling Technology, Danvers, MA, USA), anti-STAT3 (dilution 1:1000, Cat# 9139, Cell Signaling Technology, Danvers, MA, USA) and anti-phospho-STAT3 (Y705) (dilution 1:1000, Cat# 9131, Cell Signaling Technology, Danvers, MA, USA). After incubation with the primary antibodies, membranes were washed and treated for 1 h with the following secondary antibody accordingly to the source of primary antibody: Peroxidase-AffiniPure Goat Anti-mouse IgG (dilution 1:10000, Cat# 115-035-146, Jackson ImmunoResearch Labs, West Grove, PA, USA) and Peroxidase-AffiniPure Goat Anti-rabbit IgG (dilution 1:10,000, Cat# 115-035-144, Jackson ImmunoResearch Labs, West Grove, PA, USA). After this incubation, membranes were washed and developed by using Amersham ECL Western Blotting Reagent (Cat# RPN2124, GE Healthcare Bio-Sciences, Pittsburgh, PA, USA). Staining was evaluated by using C-DiGit Blot Scanner (LiCor, Lincoln, NE, USA) and quantifications of the blots were performed by using the software Image J64 (http://imagej.nih.gov/ij, NIH, USA).

### Accession to public repository of mRNA library of human breast cancer

Data were collected by using the Gene Expression Browser (GXB) via Sidra Medical and Research Center Gateway,^26^ by assessing the Nagalla Reconstituted Public Data Set,^27^ which includes the data from 1839 breast cancer patients. mRNA array data were obtained by Affymetrix.

### Data analyses and statistics

All graphics and statistical analyses were performed with software Prism 7 for Mac (GraphPad Software Inc., La Jolla, CA, USA). Student’s *t* test, one-way ANOVA followed by Tukey’s post test or two-way ANOVA followed by Sidak’s post test were used as appropriate.

## Results

### Serotonin confers proliferative advantages to MCF-7 cells affecting their metabolic profile

**Fig. 1:**
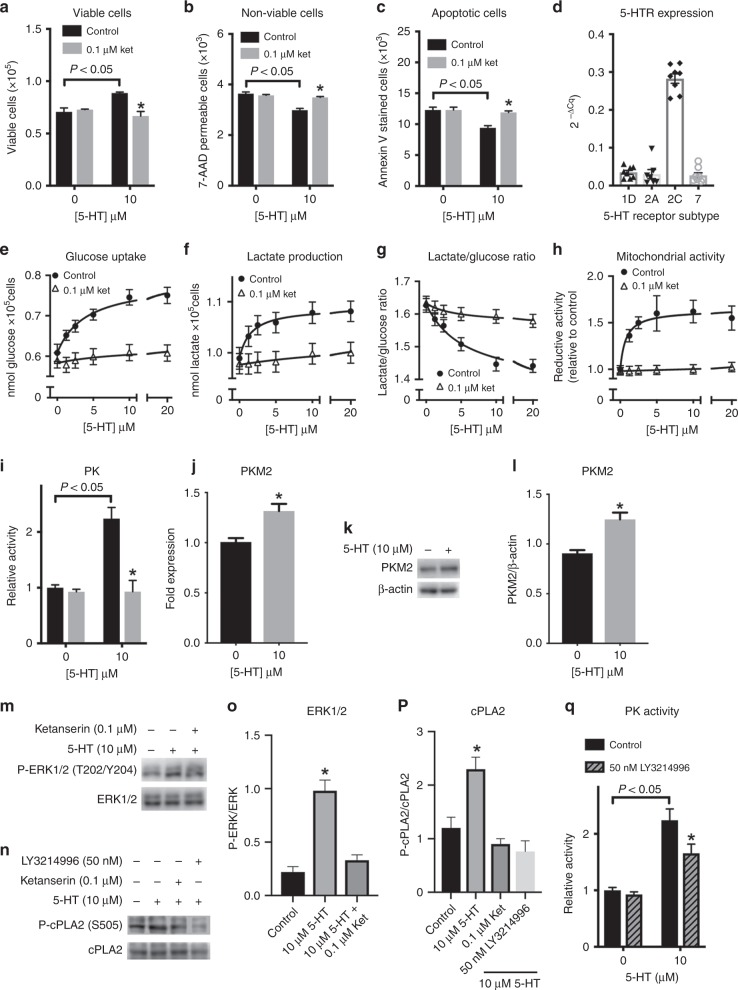
Serotonin modulates MCF-7 cells proliferation and metabolism. **a** Proliferation rate, **b** cell viability, **c** apoptotic cells, **d** 5-HT receptor expression, **e** glucose consumption, **f** lactate production, **g** lactate production/glucose consumption ratio and **h** mitochondrial activity of MCF-7 cells treated with different 5-HT concentrations in the absence or the presence of 0.1 µM ketanserin (5-HT_2A/C_ receptor antagonist). These plotted values are the mean ± S.E.M. of six independent experiments (*n* = 6). For **a**, **b**, **c** and **d**, the data were analysed by two-way ANOVA, followed by Sidak’s post test and * means *P* < 0.05 compared with the black bar of the same group. For **e**, **f** and **h**, except for the results in the absence of 5-HT, the differences between each value in the absence and in the presence of ketanserin were significant (*P* < 0.05; two-way ANOVA, followed by Sidak’s post test). For **g**, the differences were significant at the concentrations of 5, 10 and 20 µM 5-HT (*P* < 0.05; two-way ANOVA, followed by Sidak’s post test). The enzymatic activity of PK (**i**) and the levels of mRNA of PKM2 (**j**) are shown. For PK activity, plotted values are the mean ± S.E.M. of six independent experiments (*n* = 6; data were analysed by two-way ANOVA, followed by Sidak’s post test and * means *P* < 0.05 compared with the black bar of the same group). For PKM2 mRNA expression, plotted values are the mean ± S.E.M. of six independent experiments (*n* = 6; * means *P* < 0.05, Student’s *t* test). **k** Representative western blot of evaluation of PKM2 expression. **l** Quantification of the western blots of evaluation of PKM2 expression of three independent experiments (*n* = 3). Quantification (**m**) and representative western blots (**o**) of phosphorylation of ERK1/2 in MCF-7 cells treated with 5-HT and ketanserin (*n* = 3; * means *P* < 0.05 as compared with control in the absence of 5-HT, Student’s *t* test). Quantification (**o**) and representative western blots (**n**) of phosphorylation of cPLA2 in MCF-7 cells treated with 5-HT, ketanserin and LY3214996 (*n* = 3; * means *P* < 0.05 as compared with control in the absence of 5-HT, Student’s *t* test). **q** Pyruvate kinase activity in the absence or presence of 10 µM 5-HT and 50 nM LY3214996 (ERK1/2 inhibitor). The results for enzyme activity are from five independent experiments (*n* = 5, * means *P* < 0.05 as compared with control in the absence of LY3214996 and the bracket indicates *P* < 0.05 as compared with the absence of 5-HT; two-way ANOVA, followed by Sidak’s post test). Plotted results are expressed as mean ± S.E.M.

When MCF-7 cells were grown in the presence of 10 µM 5-HT, we observed a 28% increase in the number of cells after 18 h of incubation as compared with control (Fig. 1a). This treatment decreased the permeability of cancer cells to 7-AAD (Fig. 1b) and reduced the staining for Annexin V (Fig. 1c). These effects are prevented by the presence of 0.1 µM ketanserin, an antagonist of the 5-HT_2_ receptor family.^28^ To identify 5-HT receptors expressed by MCF-7 cells, we performed a qPCR for all the 5-HT receptor subtypes and found the expression of only 5-HTR_1D_, 5-HTR_2A_, 5-HTR_2C_ and 5-HTR_7_ (Fig. 1d). The 5-HTR_1D_ receptor presents a low affinity for ketanserin (Ki ≅15 µM),^29^ while 5-HT_7_ is not antagonised by the drug.^30^ Therefore, these two receptors are not playing a role on the observed effects of 5-HT on MCF-7 proliferation. That being said, we performed the experiments that follow considering the effects of 5-HT observed as a consequence of activation of 5-HTR_2A/C_ receptors only.

In addition to the proliferative and anti-apoptotic effects reported above, 5-HT promoted a dose-dependent increase in glucose consumption by MCF-7 cells (Fig. 1e). This effect was accompanied by an increase in lactate production (Fig. 1f), suggesting that the hormone is activating glucose metabolism. However, the ratio of lactate production/glucose uptake decreased as a function of the hormone concentration (Fig. 1g), indicating that the fate of increased glucose metabolism is changing as a consequence of 5-HT action. In this context, our results revealed activation of MCF-7 mitochondrial metabolism by 5-HT (Fig. 1h), which suggests that mitochondrial oxidation might be the fate of increased glucose uptake by these cells. All these effects are prevented when the 5-HT_2A/2C_ receptor antagonist, ketanserin, is present in the assays, suggesting that these receptors are triggering the response of MCF-7 cells to 5-HT (Fig. 1a–h).

### Serotonin modulates MCF-7 cells metabolic enzyme activity and expression

Next, we sought to identify the mechanism by which 5-HT is shifting MCF-7 metabolism towards oxidation. For this, we evaluated the activity of the major regulatory glycolytic enzymes, namely hexokinase (HK), phosphofructokinase (PFK) and pyruvate kinase (PK), within MCF-7 cells treated with 10 µM 5-HT. Although the three enzymes were stimulated by the hormone, the effects on HK and PFK were modest compared with the effects of 5-HT on PK activity that increased 100% upon 5-HT stimulation as shown in Fig. 1i, while the increase in HK and PFK activity was ~10% (Supplementary Fig S1A and S1B for HK and PFK, respectively). Previous work from our group indicated that 5-HT regulates PFK activity in muscle cells.^9–11^ However, the current data suggest that PK is a major target for 5-HT action within glycolysis upregulation in cancer cells. Augmented lactate formation by 5-HT treatment in MCF-7 cells may be a result of the increase in lactate dehydrogenase (LDH) activity upon 5-HT stimulation (Supplementary Fig. S1C). It is noteworthy mentioning that the increase in LDH activity is also ~10%. This effect might be related to the decreased lactate production/glucose consumption ratio in the 5-HT-treated MCF-7 cells (Fig. 1g). This possibility is related to the doubling in PK activity, which might represent a production of pyruvate greater than the capacity of its reduction by LDH. Nevertheless, glucose-6-phosphate dehydrogenase (G6PDH) activity is not affected by 5-HT, suggesting that the hormone is not interfering with the pentose-phosphate pathway (Supplementary Fig. S1D).

Glycolysis is a multimode regulated pathway, which involves different allosteric effectors, post-translational modifications and differential expression of the enzymes’ isoforms. To evaluate this latter mode of regulation, we assessed the expression of the different isoforms of HK, PFK, and PK upon 5-HT treatment of MCF-7 cells with 5-HT. Curiously, despite the modest increase in the activity of HK and PFK (shown in Supplementary Fig. S1A and S1B), the different isoforms of these two enzymes were downregulated upon 5-HT treatment. The expression of HK1 decreased approximately by 40% upon 5-HT treatment (Supplementary Fig. S1E), while the decrease in HK2 expression reached almost 70% (Supplementary Fig. S1F). This occurs in contrast to the 10% activation of this enzyme observed under the same treatment (Supplementary Fig. S1A). Similarly, the expression of the three PFK isoforms, PFKM (Supplementary Fig. S1G), PFKL (Supplementary Fig. S1H) and PFKP (Supplementary Fig. S1I) was reduced by around 10–20% upon treatment with 5-HT, despite the 10% activation of the enzyme (Supplementary Fig. S1B). The expression of the different PK isoforms upon 5-HT treatment followed a unique pattern. While there was no effect on PKL expression (Supplementary Fig. S1J), PKM1 expression decreased by 20% (Supplementary Fig. S1K) and PKM2 expression increased by 30% (Fig. 1j). This increase in PKM2 mRNA levels was also observed in the protein levels through western blot analysis (Fig. 1k, l). PKM2 represents 60% of the total PK expressed within these cells with no treatment (Supplementary Fig. S1L), which means that after 5-HT stimulation, this isoform represents nearly 80% of total PK activity within the cells (Supplementary Fig. S1M) and there is a net increase of ~15% in total PK expression. For PK activity, we observed a 2.1-fold increase upon 5-HT stimulation (Fig. 1i) and this cannot be explained solely by the increase in PK expression. In this context, it is well described that PKM2 isoform is the multiregulated isoform that can be activated by several signals,^31,32^ including ERK1/2-mediated phosphorylation of the enzyme.^33^ In addition, both 5-HT_2A_ and 5-HT_2C_ receptors activate ERK1/2 by MEK-mediated phosphorylation.^34^ Indeed, we showed that 5-HT promotes ERK1/2 phosphorylation (Fig. 1m, o), which is confirmed by evaluating the phosphorylation of ERK1/2 substrate, the cytosolic phospholipase A2 (cPLA2) on S505 (Fig. 1n, p). Moreover, 5-HT-stimulated phosphorylation of both ERK1/2 and cPLA2 is prevented by ketanserin (Fig. 1m–p), and the phosphorylation of cPLA2 is prevented by LY3214995 (Fig. 1n, p). In addition, the treatment of MCF-7 cells with LY3214996, an inhibitor of ERK1/2, partially prevented PK activation by 5-HT (Fig. 1q). To this end, we concluded that 5-HT-induced activation of PK might be due to an increased expression of the enzyme, especially PKM2 rather than the other isoforms, which makes the enzyme more responsive to ERK1/2 signalling. Yet, the only partial reversal of 5-HT-induced PK activation by ERK1/2 inhibition indicates that both increased expression of the enzyme and its activation by ERK1/2 are independently contributing to the increased PK activity induced by 5-HT on MCF-7 cells.

### Serotonin triggers mitochondrial biogenesis in MCF-7 cells

**Fig. 2:**
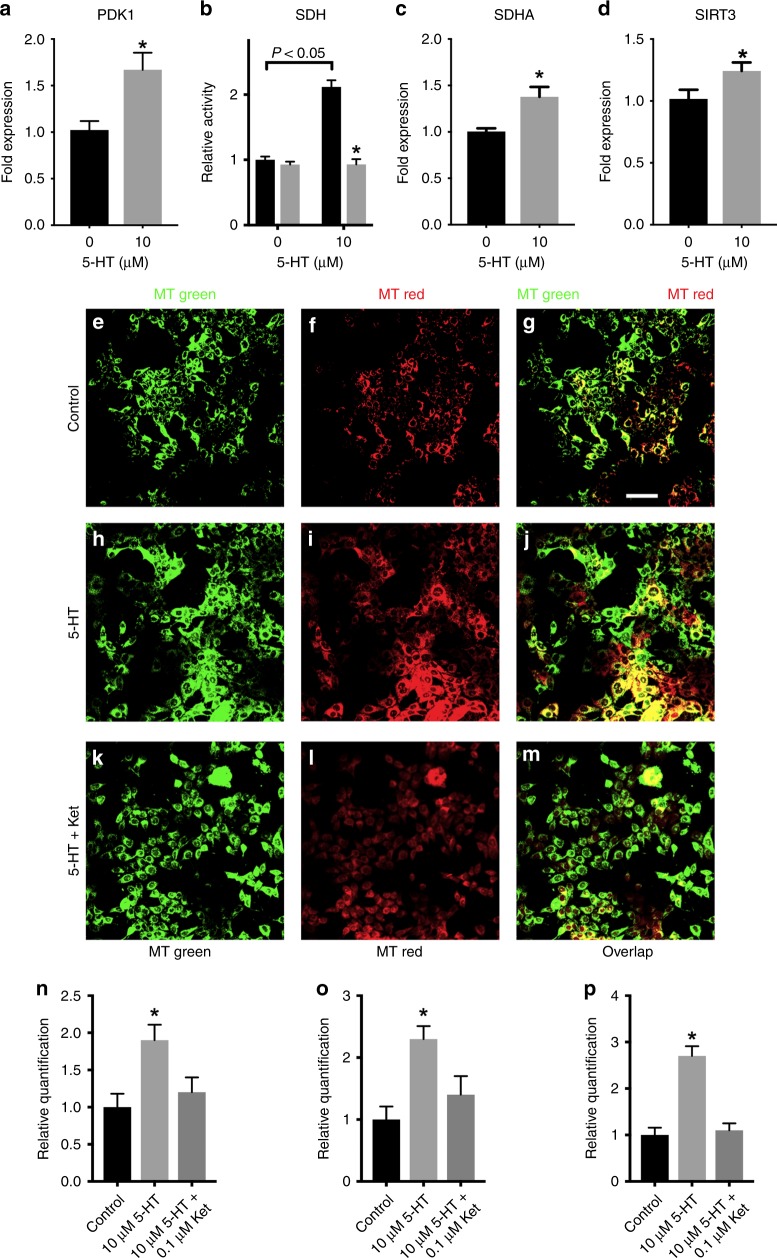
Serotonin modulates MCF-7 cells mitochondria biology. **a** Relative RNA expression of pyruvate dehydrogenase kinase 1 (PDK1), (**b**) enzymatic activity of succinate dehydrogenase (**b**), (**c**) relative RNA expression of succinate dehydrogenase A (SDHA) and (**d**) relative RNA expression of SIRT3. Plotted values are mean ± S.E.M. of seven independent experiments (*n* = 7; for **a**, **c** and **d**, * means *P* < 0.05 as compared with control in the absence of 5-HT, Student’s *t* test; for **c** * means *P* < 0.05 as compared with control in the absence of ketanserin and the bracket indicates *P* < 0.05 as compared with the absence of 5-HT; two-way ANOVA, followed by Sidak’s post test). **e**–**m** Representative confocal images of MCF-7 cells treated in the absence (**e**–**g**) or the presence of 5-HT (**h**–**j**) and 5-HT plus ketanserin (**k**–**m**), stained with MitoTracker green FM (**e**, **h**, **k**) and MitoTracker Red CMXRos (**f**, **i**, **l**). **n** Quantification of MitoTracker green FM staining. **o** Quantification of MitoTracker Red CMXRos staining. **p** Quantification of overlapping. Plotted values in panels N–P are mean ± S.E.M. of three independent experiments (*n* = 3; * means *P* < 0.05 compared with control; one-way ANOVA followed by Tukey’s post test)

The role of PKM2 in cancer has been scrutinised and reviewed by many authors, and it is clear that the expression of this PK isoform promotes cell growth and proliferation.^35–39^ Among the advantages of expression of PKM2 is the favouring of the Warburg effect over oxidation^40^ through promoting the expression of HIF-1α.^39^ Indeed, 5-HT promotes the expression of HIF-1α, as will be further shown and discussed. HIF-1α promotes the expression of pyruvate dehydrogenase kinase 1 (PDK1) that inhibits the pyruvate dehydrogenase complex (PDH).^41,42^ Therefore, we evaluated PDK1 expression in 5-HT-treated MCF-7 cells and found a 60% increase in the levels of PDK1 mRNA (Fig. 2a). Nevertheless, we observed here that 5-HT increases glucose consumption and mitochondria activity at the same time, suggesting that the fate of glucose is the oxidation. Indeed, 5-HT promotes a twofold activation of succinate dehydrogenase (SDH) activity, which is also prevented by ketanserin (Fig. 2b). This explains our aforementioned observation of the increased mitochondrial metabolism upon 5-HT stimulation of MCF-7 cells (Fig. 1h). However, even though 5-HT activates SDH, inhibition of PDH by PDK1 would avoid pyruvate oxidation in mitochondria. To investigate this, we first evaluated the SDHA expression and found that 5-HT treatment increased the mRNA levels of this enzyme by ~1.5-fold (Fig. 2c), partially explaining the enzyme activation. This result also suggests that 5-HT may be promoting mitochondrial biogenesis, and hence the increased expression of PDK1 and SDHA may be due to this phenomenon. To test this possibility, we evaluated the levels of Sirtuin 3 (SIRT3), known to promote mitochondrial biogenesis.^43^ Indeed, SIRT3 mRNA levels significantly increased upon the treatment of MCF-7 cells with 5-HT (Fig. 2d). Moreover, upon staining of 5-HT-treated MCF-7 cells with MitoTracker Green (that stains mitochondria regardless of the oxidation state)^44^ and MitoTracker deep red (that stains metabolically active mitochondria),^45^ we observed an increase in total and active mitochondria in 5-HT-treated MCF-7 cells as compared with the control, and this effect was prevented by ketanserin (Fig. 2e–p). Therefore, our results strongly suggest that the increased oxidation of pyruvate by 5-HT-treated MCF-7 cells is due to the augmented number and activity of mitochondria in these cells.

### Serotonin effects involve two distinct signalling pathways

**Fig. 3:**
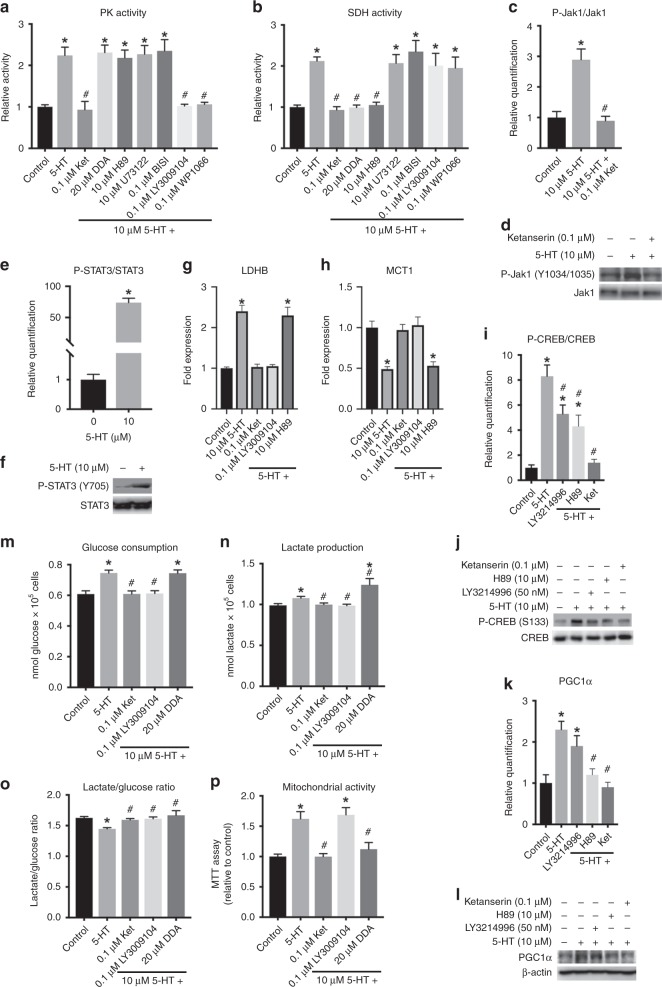
Intracellular molecular mechanisms for the serotonin actions on metabolism of MCF-7 cells. **a** Pyruvate kinase and **b** succinate dehydrogenase activities in the presence of 5-HT and several inhibitors and antagonists. Plotted values represent the means ± S.E.M. of six independent experiments (*n* = 6). *P < 0.05 as compared with control. ^#^*P* < 0.05 as compared with 5-HT. One-way ANOVA followed by Tukey’s post test. Quantification (**c**) and representative western blot (**d**) of Jak1 phosphorylation. Quantification (**e**) and representative western blot (**f**) of STAT3 phosphorylation. **g** LDHB mRNA expression. **h** MCT1 mRNA expression. **i** Quantification of CREB phosphorylation. **j** Representative western blot for CREB phosphorylation. **k** Quantification of PGC1α expression. **l** Representative western blot for PGC1α expression. For **c**–**l**, plotted values represent means ± S.E.M. of seven independent experiments (*n* = 7). * and ^#^*P* < 0.05 as compared with control or to 10 µM 5-HT, respectively (Student’s *t* test for panel E and one-way ANOVA followed by Tukey’s post test for panels **c**, **g**, **h**, **i**, **k**). **m** Glucose consumption. **n** Lactate production. **o** Lactate production/glucose consumption ratio. **p** Mitochondrial activity. For **m**–**p**, plotted values represent means ± S.E.M. of five independent experiments (*n* = 5; * *P* < 0.05 as compared with control; ^#^*P* < 0.05 as compared with 5-HT; one-way ANOVA followed by Tukey’s post test)

So far, we observed that the stimulation of MCF-7 cells with serotonin activates glycolysis and shifts the fate of pyruvate from reduction to oxidation. Glycolysis activation is primarily due to the preferential expression of PKM2, which greatly activates this enzyme. Indeed, the Warburg effect has been always associated with reduced PKM2 activity,^46^ and thus it is reasonable to postulate that increased expression and activity of this enzyme promotes an augmented oxidation of pyruvate. Moreover, the shift of the fate of pyruvate might be also due to the increased mitochondrial biogenesis observed, which is related to the higher activity of the organelle within these cells. To test how 5-HT signalling is transduced into these effects, we tested a series of inhibitors of known 5-HT signalling pathways on the activities of PK and SDH, as indicators of increased glycolysis and mitochondrial biogenesis, respectively. Our results are shown in Fig. 3a, b for PK and SDH activities, respectively, and revealed different signalling pathways involved in each mechanism. Activation of PK by 5-HT was prevented by the presence of LY3009104 and WP1066, inhibitors of Jak1 and STAT3, respectively (Fig. 3a), whereas the increased SDH activity in 5-HT-treated cells was prevented by the presence of 2′,5′-dideoxyadenosine (DDA) and H89, inhibitors of adenylyl cyclase and protein kinase A (PKA), respectively (Fig. 3b). Protein kinase C (PKC) and phospholipase C (PLC), two known transducers of 5-HT signal through 5-HT_2A/2C_ receptors are not involved in these effects of the hormone, since their inhibition by using U73122 (PLC) and BIS I (PKC) has not affected activation of PK or SDH by 5-HT (Fig. 3a, b).

To further investigate the signalling pathways linking 5-HT stimulation and the activation of Jak1/STAT3 and cAMP/PKA pathways, we evaluated the effects of 5-HT treatment on the phosphorylation of Jak1 in MCF-7 cells. We found that 5-HT promoted the phosphorylation of Jak1 on Y1034/1035 (Fig. 3c, d), a known activation site of this kinase.^47^ Moreover, this phosphorylation was prevented by the presence of 5-HT_2A/2C_ receptor antagonist, ketanserin (Fig. 3c, d). Indeed, Jak1 activation by 5-HT_2A/C_ receptor stimulation has been reported elsewhere^48^, and this kinase has been shown previously to be associated with STAT3 phosphorylation in mammary cancer cells.^49^ We confirmed this association in the current work since we also observed an increased STAT3 phosphorylation on MCF-7 cells treated with 5-HT (Fig. 3e, f). In the context of glycolytic activation, it has been demonstrated that STAT3 regulates expression of Hif-1α in cancer cells and this impacts Akt phosphorylation.^50^ Therefore, we investigated whether these events are affected by 5-HT on MCF-7 cells. Our results revealed that upon 5-HT treatment, Hif-1α expression increased in MCF-7 cells, and this effect was prevented by the presence of STAT3 inhibitor, WP1066 (Supplementary Fig. S2A, S2B). Similar effects were observed for Akt phosphorylation on T308 (Supplementary Fig. S2C, S2D), revealing that STAT3 activation by Jak1 upon 5-HT treatment of MCF-7 cells increases both the expression of Hif-1α and phosphorylation of Akt. These two events are directly involved in activation of glycolysis and may partially explain the increased glucose consumption by 5-HT-treated MCF-7 cells. Hif-1α, for instance, is directly involved in the expression of PDK1, by explaining the results presented in Fig. 2a. Moreover, STAT3-induced Akt phosphorylation triggers the expression of PKM2 in cancer cells,^51^ which also explains our findings. Nonetheless, these results do not explain the decrease in lactate production/glucose consumption ratio or the increased mitochondrial biogenesis. However, it has been reported that STAT3-activated Akt triggers the expression of lactate dehydrogenase (LDH) B, an isoform of lactate dehydrogenase responsible for the mitochondrial oxidation of lactate into pyruvate.^52^ Therefore, we evaluated the expression of the two LDH isoforms, A and B, as well as the plasma membrane lactate transporter, MCT1. We found a slight reduction in LDHA expression (Supplementary Fig. S2E) and a substantial increase in LDHB expression (Fig. 3g). Besides, MCT1 expression was reduced upon 5-HT treatment (Fig. 3h), suggesting a difficult externalisation of lactate by the cells. These effects on LDHA, LDHB and MCT1 were prevented by ketanserin and Jak1 inhibitor LY3009104, supporting the involvement of 5-HTR_2A/C_ and Jak1/STAT3 on this process (Fig. 3g, h; Supplementary Fig. S2E). Moreover, PKA inhibitor H89 did not alter the effects of 5-HT on LDHA, LDHB and MCT1 mRNA levels (Fig. 3g, h; Supplementary Fig. S2E). Cytosolic formed lactate might be taken up by mitochondria where it is oxidised by the increased LDHB within this organelle. This last effect is related to the increased mitochondria biogenesis observed in the current work. To assess this effect, we studied the effects of 5-HT on the phosphorylation of CREB on S133, a site for PKA action on this transcription factor.^53^ We observed an increased phosphorylation of CREB on S133 upon 5-HT treatment of MCF-7 cells, an effect fully prevented by ketanserin (Fig. 3i, j). The phosphorylation of CREB on S133 can be promoted by PKA and ERK1/2, which we have demonstrated to be activated by 5-HT (Fig. 1m–o). However, ERK1/2 inhibitor LY3214996 only partially prevented the phosphorylation of CREB on S133 promoted by 5-HT (Fig. 3i, j). PKA inhibitor H89 also partially prevented 5-HT-induced CREB phosphorylation, confirming that 5-HT is promoting PKA activation in MCF-7 cells. Since PKA is reported to promote the expression of PGC1α, a nodal regulator of mitochondrial biogenesis,^54^ via CREB phosphorylation, we investigated the expression of this coregulator. Indeed, 5-HT treatment increased the expression of PGC1α in MCF-7 cells (Fig. 3k, l). This effect was not significantly affected by the ERK1/2 inhibitor, LY324996, but was prevented by both H89 and ketanserin, a PKA inhibitor and a 5-HTR_2A/C_ antagonist, respectively (Fig. 3k, l). Altogether, these results strongly suggest that the effects of 5-HT promoting glucose consumption and mitochondrial biogenesis occur through two distinct signalling pathways, involving a Jak1/STAT3-induced glycolysis and cAMP/PKA-induced mitochondrial biogenesis. It is important to notice that U73122 and BIS I, inhibitors of phospholipase C and protein kinase C, respectively, do not interfere with the effects of 5-HT on neither PK nor SDH activities, supporting the notion that these signal transducers are not involved in the effects reported in the current work.

### JAK1/STAT3 activates glycolysis while cAMP/PKA triggers mitochondrial biogenesis

Aiming to prove that Jak1/STAT3 and cAMP/PKA pathways are distinctly involved in 5-HT-induced MCF-7 glucose metabolism, we treated the cells with the hormone in the absence and the presence of the Jak1 inhibitor, LY3009104, and the adenylate cyclase inhibitor, DDA, and evaluated glucose consumption, lactate production and mitochondrial activity. The results showed that the effects of 5-HT on glucose consumption (Fig. 3m), lactate production (Fig. 3n) and lactate production/glucose consumption ratio (Fig. 3o) were completely hindered by LY3009104, similar to ketanserin. DDA did not affect the stimulation of glucose consumption promoted by 5-HT on MCF-7 cells (Fig. 3m). However, the effects of 5-HT on lactate production by MCF-7 cells were enhanced in the presence of DDA (Fig. 3n). Moreover, both the Jak1 and the adenylate cyclase inhibitors reverted the effects of 5-HT on the ratio of lactate production and glucose consumption (Fig. 3o). In addition, by evaluating the effects of these inhibitors on 5-HT-stimulated mitochondrial activity of MCF-7 cells, we observed that LY3009104 did not alter the effects of 5-HT, while DDA prevented these effects (Fig. 3p). These results corroborate that the activation of glycolysis by 5-HT on MCF-7 cells is mediated by Jak1/STAT3 signalling, while the augmented mitochondrial activity is mediated by cAMP/PKA. The fact that DDA enhances the effects of 5-HT on lactate production and reverses the effects of the hormone on the ratio between lactate production and glucose consumption confirms that the increased mitochondrial activity is responsible for the shift in the fate of pyruvate from fermentative to oxidative metabolism.

We have previously observed that STAT3 is involved in the 5-HT activation of glycolysis in skeletal muscle,^11^ but in this case, the hormone target was PFK, as it was upregulated upon stimulation with 5-HT. In the current work, the small activation of PFK upon 5-HT treatment (Supplementary Fig. S1B) was also reversed by LY3009104 and WP1066 (data not shown), showing a similar mechanism to those reported for skeletal muscle.^11^ Conversely, cAMP/PKA have been reported to promote mitochondrial biogenesis.^55^ However, to the best of our knowledge, this is the first study to report that 5-HT is associated with increased PK activity and mitochondrial biogenesis, by decreasing the Warburg effect in a cancer cell. It is important to mention that although 5-HT is decreasing the Warburg effect, it is also promoting proliferative effects on MCF-7 cells, by increasing cell viability and decreasing apoptosis (Fig. 1a–c). Indeed, it has been reported that reversal of the Warburg effect followed by an increased oxidative phosphorylation presents minimal impact on tumour viability and growth.^56–59^ This might be the case of MCF-7 cells treated with 5-HT, since mitochondria metabolism is upregulated by the hormone (Figs. 2, 3p).

### Significance of the results for breast cancer

**Fig. 4:**
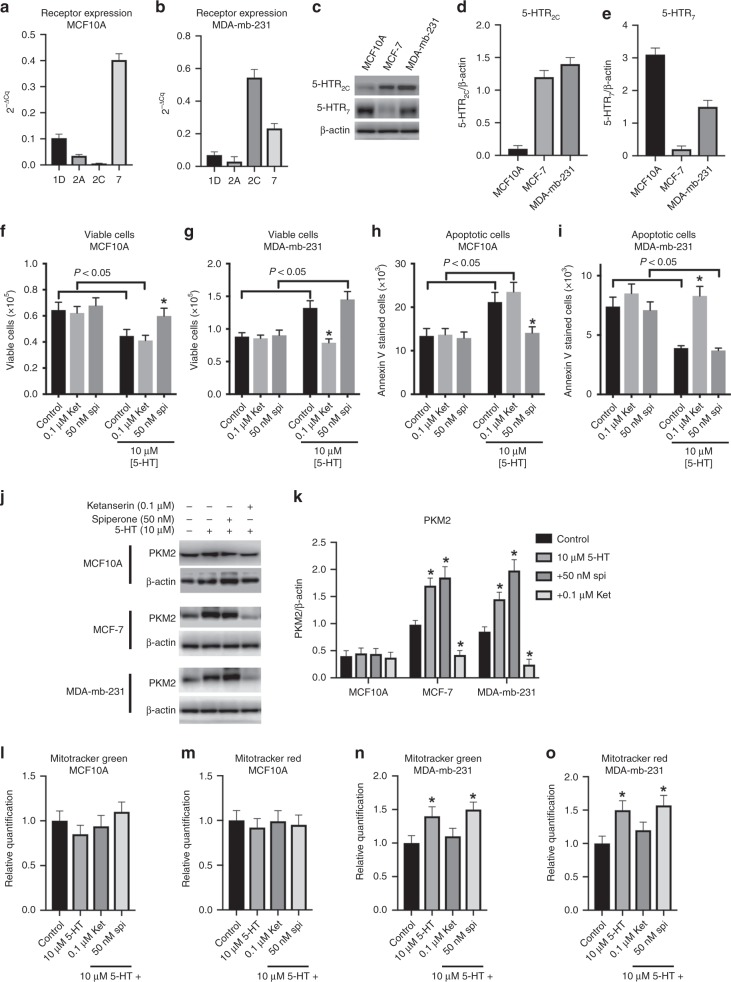
Effects of 5-HT on MCF10A and MDA-mb-231 cells biology. Expression pattern of 5-HT receptors was evaluated by qPCR in MCF10A (**a**) and MDA-mb-231 (**b**) cells. Representative western blots for 5-HTR_2C_ and 5-HTR_7_ on MCF10A. MCF-7 and MDA-mb-231 cells (**c**). Relative quantification of western blots for 5-HTR_2C_ (**d**) and 5-HTR_7_ (**e**) in comparison with the loading control β-actin (*n* = 3). Cell viability analysis of MCF10A (**f**) and MDA-mb-231 (**g**) cells (*n* = 3; * means *P* < 0.05 as compared with control in the presence of 5-HT or the absence of ketanserin or spiperone, and the bracket indicates *P* < 0.05 as compared with the absence of 5-HT; two-way ANOVA, followed by Sidak’s post test). Apoptosis analysis of MCF10A (**h**) and MDA-mb-231 (**i**) cells (*n* = 3; * means *P* < 0.05 as compared with control in the presence of 5-HT or the absence of ketanserin or spiperone, and the bracket indicates *P* < 0.05 as compared with the absence of 5-HT; two-way ANOVA, followed by Sidak’s post test). Representative western blots (**j**) and quantification (**k**) of PKM2 expression in the MCF10A, MCF-7 and MDA-mb-231 cell lines (*n* = 3. * means *P* < 0.05 as compared with control for each cell line; one-way ANOVA, followed by Tukey’s post test). MitoTracker Green (**l**) and MitoTracker Red (**m**) labelling of MCF10A cells (*n* = 3; one-way ANOVA, followed by Tukey’s post test). MitoTracker Green (**n**) and MitoTracker Red (**o**) labelling of MDA-mb-231 cells (*n* = 3. * means *P* < 0.05 as compared with control for each cell line; one-way ANOVA, followed by Tukey’s post test)

Aimed to evaluate whether the results obtained are relevant to breast cancer biology, we tested the effects of 5-HT on the non-tumorigenic human breast cell line MCF10A and on the breast cancer metastatic cell line MDA-mb-231. It was previously reported that MCF-7, MCF10A and MDA-mb-231 have different patterns of 5-HT receptor expression.^8^ Here, we confirmed these results showing that MCF10A primarily expresses 5-HTR_7_, with very low levels of 5-HTR_2A_ and 5-HTR_2C_ (Fig. 4a), and MDA-mb-231 majorly expresses 5-HTR_2C_ with intermediate levels of 5-HTR_7_ (Fig. 4b). These data contrast with the pattern of 5-HT receptors expressed by MCF-7 cells, which primarily express 5-HTR_2C_, by presenting residual expression levels of the 5-HTR_7_ (Fig. 1d). The expression patterns of 5-HTR_2C_ and 5-HTR_7_ on the three cell lines used here were confirmed by measuring the proteins levels by western blot (Fig. 4c–e). This difference in 5-HT receptor expression might be responsible for the antagonistic effects of 5-HT on MCF10A, as compared with the two tumour cell lines. For instance, 5-HT decreased the viability of MCF10A cells (Fig. 4f) but increased the viability of MDA-mb-231 (Fig. 4g). Indeed, the effects of 5-HT on MCF10A were prevented by 50 nM spiperone, a 5-HTR_7_ antagonist, but not by ketanserin (Fig. 4f). On the other hand, spiperone was ineffective on preventing 5-HT effects on MDA-mb-231, while these effects were fully prevented by ketanserin (Fig. 4g). Similarly, 5-HT induced apoptosis on MCF10A cells (Fig. 4h), while it prevented this phenomenon on MDA-mb-231 (Fig. 4i), where the effects on the former and the latter cell lines were prevented by spiperone and ketanserin, respectively (Fig. 4h, i for MCF10A and MDA-mb-231, respectively). In addition, the effects of 5-HT on the expression of PKM2 of MCF-7 cells are similar in MDA-mb-231 cells (Fig. 4j). The treatment of these cells with 5-HT increased the expression of PKM2 (Fig. 4j, k), which we have correlated to an increased glycolytic flux within these cells. This effect was prevented by ketanserin, but not by spiperone (Fig. 4j, k). On the other hand, 5-HT did not affect the expression levels of PKM2 in MCF10A cells (Fig. 4j, k). Similarly, 5-HT did not affect neither the number nor the polarisation of mitochondria in MCF10A cells, as evaluated by MitoTracker Green (Fig. 4l) and MitoTracker Red (Fig. 4m), respectively. However, the effects of 5-HT on MDA-mb-231 were similar to those shown for MCF-7 (Fig. 2), increasing both the number (Fig. 4n) and the polarisation of mitochondria (Fig. 4o). These effects were prevented by ketanserin but not by spiperone, supporting the notion that they are mediated by 5-HTR_2A/C_ receptors.

**Fig. 5:**
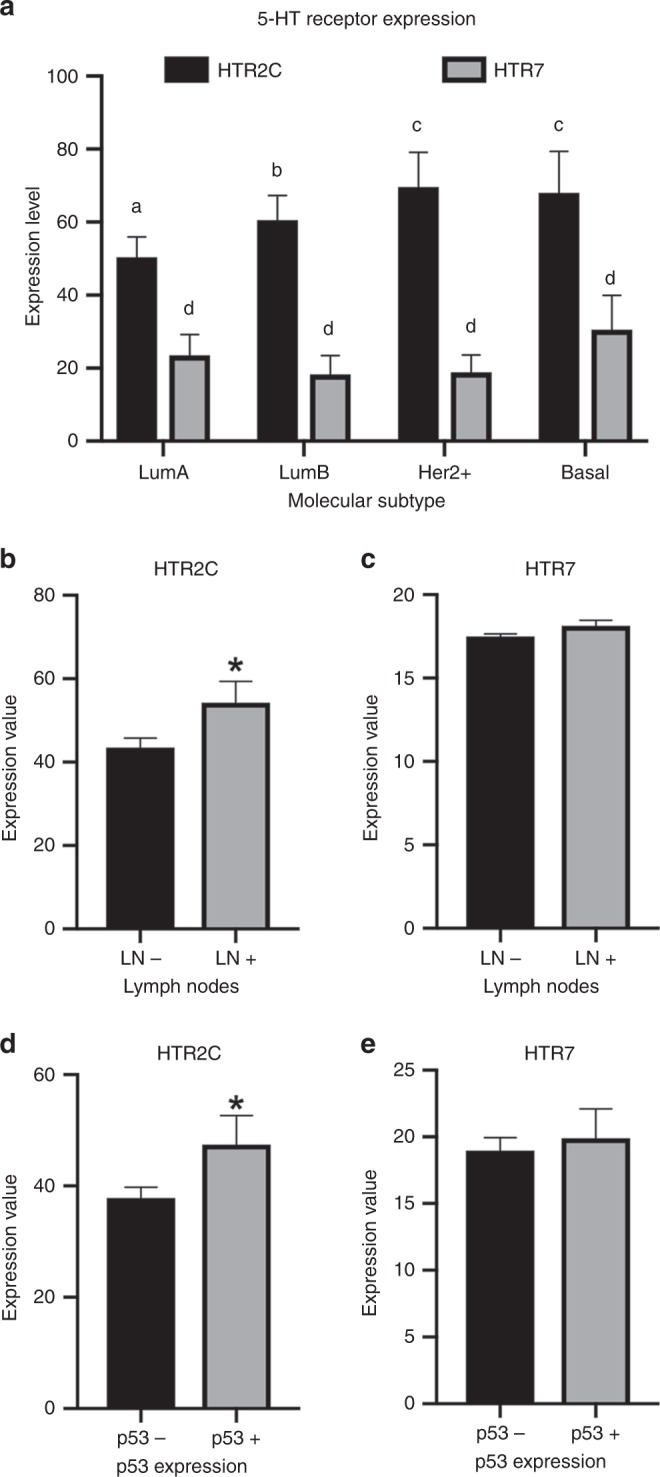
Correlation of the expression patterns of 5-HTR_2C_ and 5-HTR_7_ and prognosis markers in breast cancers. Data were collected by using the Gene Expression Browser (GXB) via Sidra Medical and Research Center Gateway, by assessing the Nagalla Reconstituted Public Data Set,^27^ which includes the data from 1839 breast cancer patients. mRNA array data were obtained by Affymetrix. **a** Correlation of 5-HTR_2C_ and 5-HTR_7_ expression and the IMS classification of the breast cancer (*n* = 398; different letters superscript means *P* < 0.05 as compared with the other values; two-way ANOVA, followed by Sidak’s post test). **b**, **c** Comparison of 5-HTR_2C_ and 5-HTR_7_ expression in lymph-node-negative (LN−) or lymph-node-positive (LN + ) primary breast cancer (*n* = 532; * means *P* < 0.05; Student’s *t* test). **d**, **e** Comparison of 5-HTR_2C_ and 5-HTR_7_ expression in p53-negative (p53−) or p53-positive (p53 + ) primary breast cancer (*n* = 275; * means *P* < 0.05; Student’s *t* test)

Aimed to investigate the expression pattern of 5-HTR_2C_ and 5-HTR_7_ on different human breast cancers, we assessed the Gene Expression Browser (GXB), via Sidra Medical and Research Center Gateway, by assessing the Nagalla Reconstituted Public Data Set,^27^ which includes the data from 1839 breast cancer patients.^26^ Breast tumours can be classified according to the intrinsic molecular subtypes (IMS), which takes into account the expression patterns of important receptors related to breast cancer progression and physiology, i.e., the oestrogen receptor (ER), progesterone receptor (PR) and human epidermal growth factor receptor-type 2 (HER2).^60^ According to IMS, breast cancers are classified as Luminal A (LumA; ER + , PR + , HER2−), Luminal B (LumB; ER + , PR + HER2 + ), HER2 + (ER−, PR−, HER2 + ) and Basal or triple negative (ER−, PR−, HER2−).^60^ This classification helps to choose the therapeutic strategy and also indicate the therapy prognosis, where LumA has a good prognosis, LumB an intermediate prognosis and HER2 + and Basal present a poor prognosis, due to the responsiveness to the available chemotherapy and the aggressivity of the tumours.^60^ Curiously, 5-HTR_2C_ expression increases among these breast cancer subtypes as the prognosis worsens, i.e., the expression levels are such as LumA < LumB < HER2 + and Basal (Fig. 5a). On the other hand, 5-HTR_7_ expression did not vary among breast cancers classified according to the IMS (Fig. 5a). Similarly, dividing the breast cancer between those whose patients showed cancer cells within their lymph nodes (LN + ) or not (LN−), we observed a higher expression of 5-HTR_2C_ in LN + , as compared with LN− (Fig. 5b), while 5-HTR_7_ expression was not different between these two groups (Fig. 5c). The exact same result was obtained separating the breast cancers between those positive and negative for p53 (p53 + and p53−, respectively), where 5-HTR_2c_ was upregulated in p53 + , as compared with p53− cancer (Fig. 5d), while 5-HTR_7_ remained unaltered between the two groups (Fig. 5e). Evaluation of lymph nodes and p53 expression is extremely important for breast cancer patients since these characteristics are directly associated with the cancer aggressiveness, the prognosis, the recurrence rate and survival.^60–62^ Our data here strongly indicate that the higher the 5-HTR_2C_ expression, the worse is the prognosis for the breast cancer patients. Since we characterised that 5-HT signalling via this receptor alters breast cancer cell metabolism towards highly glycolytic and highly oxidative, through upregulation of PKM2 and mitochondria biogenesis, we propose that these characteristics are directly associated with breast cancer aggressiveness and poor prognosis.

## Discussion

One of the hallmarks of cancer cells is a deregulated energy metabolism including the so-called aerobic glycolysis, or Warburg effect, where most of the consumed glucose is converted into lactate regardless of the oxygen supply to these cells.^63^ In fact, aerobic glycolysis is required for fast-growing cells due to the rapid supply of ATP and the diversion of glycolysis intermediates into various biosynthetic pathways.^63^ However, there are growing evidences exemplifying that aerobic glycolysis is not constitutively activated in cancer cells that constantly shift to oxidative metabolism, which contribute to cancer progression and metastasis.^64^ This shift involves the mitochondrial oxidation of pyruvate, decreasing the ratio of lactate formation.^64^ In the current paper, we show that serotonin confers proliferative advantages to breast cancer cells, which involves not only stimulation of glucose metabolism but also a shift from fermentative to oxidative metabolism. This effect is observed only in the cancer cells and not in the non-tumorigenic breast cell line, MCF10A.

Our data show that serotonin is signalling a proliferative advantage to breast cancer cells, by increasing the rate of cell proliferation and decreasing programmed cell death. Similar results have been published earlier by Sonier et al., where authors reported that 5-HT-treated MCF-7 cells proliferate faster than control, an effect that was attributed to the expression of 5-HTR_2A_ by these cells.^65^ Here, through q-RT-PCR, we screened MCF-7 cells for the expression of several 5-HT receptors and found expression of only 5-HTR_1D_, 5-HTR_2A_, 5-HTR_2C_ and 5-HTR_7_ (Fig. 1d). Among these four 5-HT receptors, 5-HTR_2C_ demonstrated the highest expression by MCF-7 cells (at least ten times more expressed than the other three receptors, Fig. 1d). Similar results were reported by Pai et al. who also showed that MCF-7 cells express 5-HTR_1E_, 5-HTR_1F_ and 5-HTR_2B_,^8^ but these authors used end-point PCR, which might have detected residual mRNA levels. Moreover, the proliferative effects of 5-HT on MCF-7 cells were hindered in the presence 0.1 µM ketanserin, which is at least ten times the Ki for 5-HTR_2A_ and 5-HTR_2C_^28^ (Fig. 1a–c), by corroborating the results published by Sonier et al.^65^ In their paper, Sonier et al. have attributed the ketanserin effects to the antagonism of the drug on 5-HTR_2A_ subtype.^65^ However, since ketanserin is also a 5-HTR_2C_ antagonist^66^ and due to the predominance of 5-HTR_2C_ over 5-HTR_2A_, we believed that the former is the major receptor responsible for 5-HT actions on MCF-7 cells. Nevertheless, we cannot discard a possible transduction by 5-HTR_2A_ as proven by Sonier et al.,^65^ since both are present in these cells (Fig. 1d) and represent similar affinities and responsiveness to 5-HT and ketanserin.^28^

Altogether, our data indicate opposite effects of 5-HT on cancer and non-cancer cells. While conferring proliferative advantages to both tumoural cells (MCF-7 and MDA-mb-231), 5-HT decreased the non-cancer MCF10A proliferation and promoted apoptosis of this cell line. Indeed, these results are in accordance with previous work demonstrating the role of 5-HT on the evolution and regression of mammary cells, especially during lactation.^8,67,68^ The effects of 5-HT reported on the mammary gland also include modulations of glucose transport and metabolism, supporting our finding here.^68^ Accordingly, the differential effects of 5-HT on the mammary gland evolution might be due to the pattern of 5-HT receptor expression,^8^ where the 5-HTR_7_ plays a role in the involution of the mammary gland possibly due to promoting cell apoptosis.^69^ Conversely, our data indicated that 5-HTR_2A/C_ is related to promoting cells proliferation and inhibiting the apoptosis process, by conferring proliferative advantages to these cells. These data are strongly supported by another study where authors have proposed the proliferative action of 5-HT on breast cancer cells via 5-HTR_2A_.^65^ Pai et al. also reported that the pattern of 5-HT receptor expression varies with the type of breast tumours.^8^ Indeed, these authors also found expression of other 5-HT receptor subtypes among the same cell lines that we used here; however, their analyses were performed by end-point PCR, which detects also negligible levels of mRNA.^8^

**Fig. 6:**
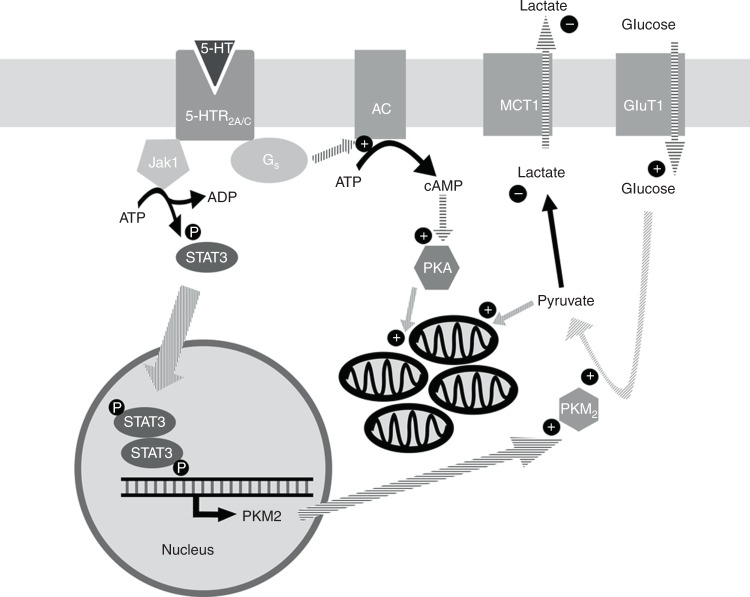
Schematic representation for the mechanism of action of serotonin on MCF-7 metabolism

Conclusively, we propose that 5-HT, through 5-HT_2A/2C_ receptors, phosphorylates Jak1, which in turn phosphorylates STAT3 promoting ERK1/2 activation, Akt phosphorylation and Hif-1α expression. Together, these effects promote glucose consumption by the preferential expression of PKM2. Simultaneously, 5-HT_2A/2C_ receptor activation by 5-HT triggers adenylate cyclase, by activating PKA that promotes the expression of PGC1α. Finally, this coactivator induces mitochondrial biogenesis, thus shifting the fate of pyruvate formed due to increased glycolysis towards oxidation via mitochondrial metabolism (Fig. 6). Consequently, the breast cancer cells become more proliferative and less susceptible to apoptosis, being able to form more aggressive cancers.

## Supplementary information

Supplementary Material

## Data Availability

All pertinent data to support this study are included in the paper and supplementary files. Further data supporting the findings are available upon request.
