# Supplementary material for: Serotonin activates glycolysis and mitochondria biogenesis in human breast cancer cells through activation of the Jak1/STAT3/ERK1/2 and adenylate cyclase/PKA, respectively
Source: Br J Cancer. 2019 Dec 10;122(2):194–208. doi: 10.1038/s41416-019-0640-1 (PMC7052254; doi:10.1038/s41416-019-0640-1)
Supplement: Supplementary file 1 — Supplementary Material [file 41416_2019_640_MOESM1_ESM.pdf]

Supplementary material.

Enzymatic activities

The enzymatic activities were evaluated after the treatment for 24 hours of cells seeded in 96-well plates and grown to 70% confluency. Following treatment, the medium was removed, and cells were lysed with 100 µl of 10 mM phosphate buffer (pH 7.4) by pipetting up and down several times. The cell-free homogenates were used for evaluating the enzymes' activities. Hexokinase activity was assessed in a reaction medium containing 50 mM Tris-HCl (pH 7.4), 5 mM $MgCl_2$, 1 mM glucose, 1 mM ATP, 150 mM KCl, 0.2 mM $NADP^+$, and 0.2 mU/ml glucose-6-phosphate dehydrogenase. Phosphofructokinase activity was assessed in a reaction medium containing 50 mM Tris-HCl (pH 7.4), 1 mM $MgCl_2$, 1 mM $(NH_4)_2SO_4$, 1 mM fructose-6-phosphate, 0.1 mM ATP, 150 mM KCl, 0.2 mM NADH, 0.2 mU/ml aldolase, 0.4 mU/ml triosephosphate isomerase, and 0.2 mU/ml α-glycerophosphate dehydrogenase. Pyruvate kinase activity was assessed in a reaction medium containing 50 mM Tris-HCl (pH 7.4), 1 mM $MgCl_2$, 1 mM phosphor(enol)pyruvate, 1 mM ADP, 150 mM KCl, 0.2 mM NADH, and 2 mU/ml lactate dehydrogenase. Lactate dehydrogenase activity 50 mM Tris-HCl (pH 7.4), 1 mM $MgCl_2$, 1 mM pyruvate, and 0.2 mM NADH. Glucose-6-phosphate dehydrogenase activity was assessed in a reaction medium containing 50 mM Tris-HCl (pH 7.4), 5 mM $MgCl_2$, 1 mM glucose-6-phosphate, 150 mM KCl, and 0.2 mM $NADP^+$. Reactions were started by adding 10 µl of the cell-free homogenates and NAD(P) reduction/oxidation was followed spectrophotometrically at 340 nm. The slopes of the curves were used to determine the enzymatic activities. Succinate dehydrogenase was assessed in a reaction medium containing 50 mM Tris-HCl (pH 7.4), 5 mM $MgCl_2$, 12 mM diethyl succinate, 0.2 mM 1-methoxy-5-methylphenazinium methyl sulphate, and 1.2 mM nitro-blue-tretazolium (NBT), in the absence and the presence of 12 mM malonate (a competitive inhibitor of succinate dehydrogenase). Reaction was started by adding 10 µl cell-free homogenate and the reduce NBT was evaluated spectrophotometrically at 600 nm. Succinate dehydrogenase activity was calculated by the slope of the curve.

Quantitative PCR (qPCR)

For RNA extraction, cells were seeded in 6-well plates (10^5 cells/well) and grown to 70% confluency. Then the media were removed, and cells were treated according to the experiments. After the treatments, the media were removed and 500 µl of Trizol reagent (ThermoFischer, Carlsbad, CA, USA) was added to each well, followed by homogenization by up and down pipetting. After this, RNA extraction was performed following the directions of the reagent. For cDNA synthesis, the High-capacity cDNA Reverse Transcription Kit (ThermoFischer, Carlsbad, CA, USA) was used. For qPCR, the GoTaq qPCR Master Mix (Promega, Fitchburg, WI, USA) was used and reaction was performed in a QuantStudio 5 (ThermoFischer, Carlsbad, CA, USA). The program for all amplifications was 2 min at 95 ^oC followed by 40 cycles of 15 s at 95 ^oC and 1 min at 60 ^oC. A dissociation curve was performed at the end of the experiment and dissociation peak was analyzed. The fold expression was calculated by the $2^{-\Delta\Delta Ct}$ method, as described previously [21]. Expression levels are represented by the $2^{-\Delta Ct}$ method. *CCSER2* expression was used as reference gene (housekeeper) since its expression did not vary upon any of the used treatments (data not shown). Primers were designed using Primer-blast tool [22] and all qPCR conditions were optimized following international standards [23]. The primers used are described on Table 1.

Western blotting

For Western blot, cells were seeded in 6-well plates (10^5 cells/well) and grown to 70% confluency. Then the media were removed, and cells were treated according to the experiments. After the treatments, the media were removed and a mild-RIPA buffer [24] supplemented with protease inhibitor cocktail (Sigma-Aldrich, St. Louis, MO, USA) was added for total protein extraction. Protein extracts were submitted to SDS-PAGE (8% gels) [25], followed by overnight transfer to nitrocellulose membranes at 30 V. Membranes were stained with Ponceau S, processed and de-stained by washing with distilled water. Then, the membranes were incubated overnight with the following antibodies: anti-5-HT2C receptor (dilution 1:1000, Cat# ab197776, Abcam, Cambridge, UK), anti-5-HT7 receptor (dilution 1:1000, Cat# ab61562, Abcam, Cambridge, UK), anti-ß-actin (dilution 1:1000, Cat# 4967, Cell Signaling Technology, Danvers, MA, USA), anti-Akt (dilution 1:1000, Cat# 9272, Cell Signaling Technology, Danvers, MA, USA), anti-phospho-Akt (T308) (dilution 1:1000, Cat# 9275, Cell Signaling Technology, Danvers, MA, USA), anti-CREB (dilution 1:1000, Cat# 9197, Cell Signaling Technology, Danvers, MA, USA), anti-phospho-CREB (S133) (dilution 1:1000, Cat# 9196, Cell Signaling Technology, Danvers, MA, USA), anti-Hif-1α (dilution 1:1000, Cat# NB 100-449, Novus Biologicals, Centennial, CO, USA), anti-Jak1 (dilution 1:1000, Cat# 3332, Cell Signaling Technology, Danvers, MA, USA), anti-phospho-Jak1 (Y1034/1035) (dilution 1:1000, Cat# 3331, Cell Signaling Technology, Danvers, MA, USA), anti-PGC1α (dilution 1:1000, Cat# 2178, Cell Signaling Technology, Danvers, MA, USA), anti-PKM2 (dilution 1:1000, Cat# D78A4, Cell Signaling Technology, Danvers, MA, USA), anti- cPLA2 (dilution 1:1000, Cat# 2832, Cell Signaling Technology, Danvers, MA, USA), anti-phospho-cPLA2 (S505) (dilution 1:1000, Cat# 2831, Cell Signaling Technology, Danvers, MA, USA), anti-STAT3 (dilution 1:1000, Cat# 9139, Cell Signaling Technology, Danvers, MA, USA), and anti-phospho-STAT3 (Y705) (dilution 1:1000, Cat# 9131, Cell Signaling Technology, Danvers, MA, USA). After incubation with the primary antibodies, membranes were washed and treated for 1 hour with the following secondary antibody accordingly to the source of primary antibody: peroxidase-affinipure goat anti-mouse IgG (dilution 1:10000, Cat# 115-035-146, Jackson ImmunoResearch Labs, West Grove, PA, USA) and peroxidase-affinipure goat anti-rabbit IgG (dilution 1:10000, Cat# 115-035-144, Jackson ImmunoResearch Labs, West Grove, PA, USA). After this incubation, membranes were washed and developed using Amersham ECL Western Blotting Reagent (Cat# RPN2124, GE Healthcare Bio-Sciences, Pittsburg, PA, USA). Staining was evaluated using C-DiGit Blot Scanner (LiCor, Lincoln, NE, USA) and quantifications of the blots were performed using the software Image J64 (http://imagej.nih.gov/ij NIH, USA).

Data analyses and statistics

All graphics and statistical analyses were performed with software Prism 7 for Mac (GraphPad Software Inc, La Jolla, CA, USA). Student's *t*-test, one-way ANOVA followed by Tukey's post-test, or two-way ANOVA followed by Sidak's post-test were used as appropriated.

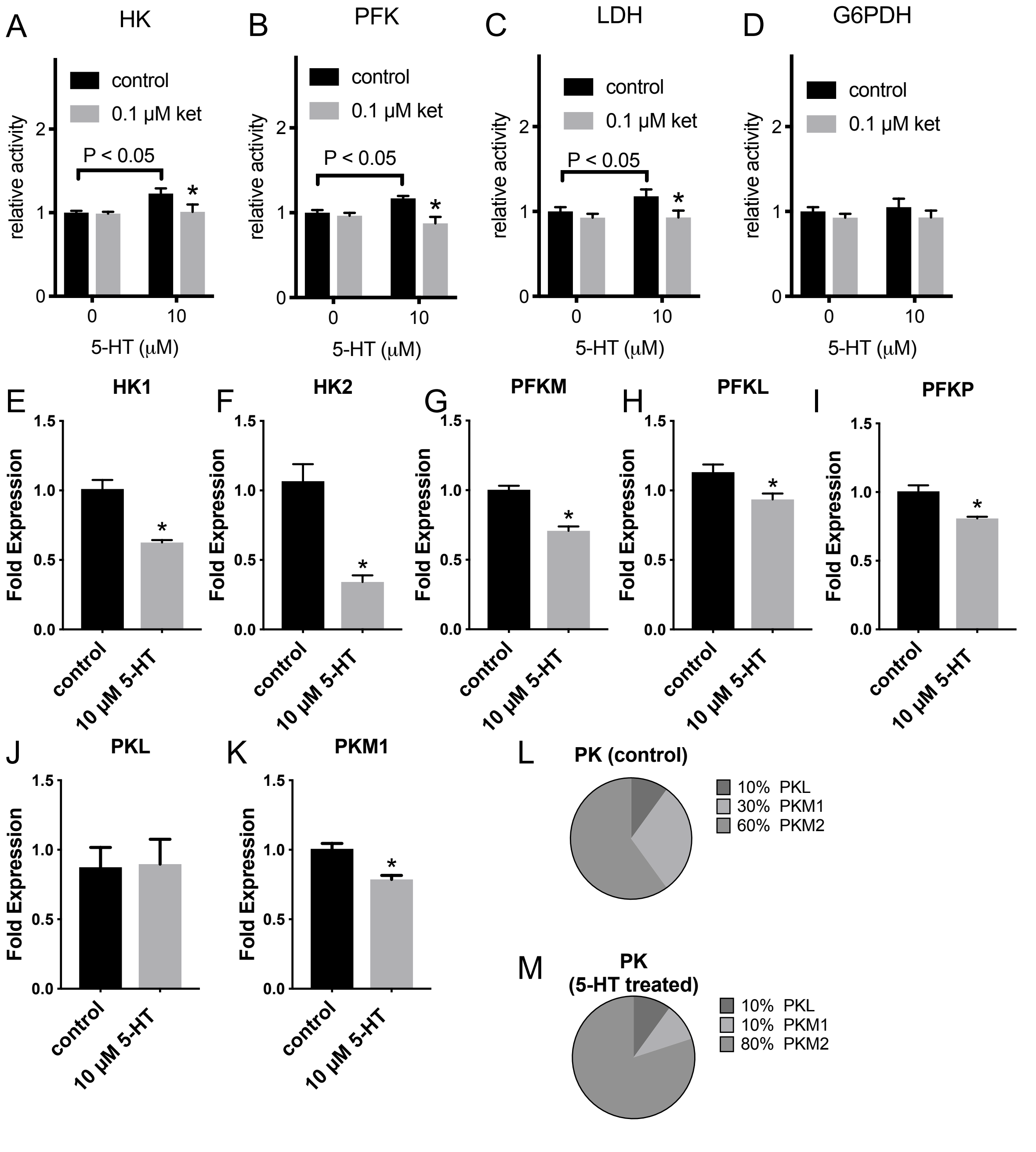

Figure S1

Fig. S1. Serotonin modulates key enzymes in MCF-7 cells. Enzymatic activities of (A) hexokinase (HK), (B) phosphofructokinase (PFK), (C) lactate dehydrogenase (LDH), and (D) glucose-6-phosphate dehydrogenase (G6PD) in the absence and the presence of 10 µM 5-HT and 0.1 µM ketanserin (5-$HT_{2A/C}$ receptor antagonist). Plotted values represent the means ± S.E.M. of 6 independent experiments (n = 6). The significant differences between the results in the absence and in the presence of ketanserin at each 5-HT concentration are indicated with star (*) and between the absence and the presence of 5-HT are indicated by the brackets ($P < 0.05$; two-way ANOVA, followed by Sidak's post-test). Expression of mRNA of (E) HK1, (F) HK2, (G) PFKM, (H) PFKL, (I) PFKP, (J) PKL and (K) PKM1. All q-RT PCR results were obtained using 7 biological independent experiments (n = 7) and the stars (*) signify

P<0.05 as compared to control in the absence of 5-HT (Student's *t*-test). Pie graphs showing the comparison between the relative expression of PKM2 before (K) and after (L) the treatment with 10 μM 5-HT.

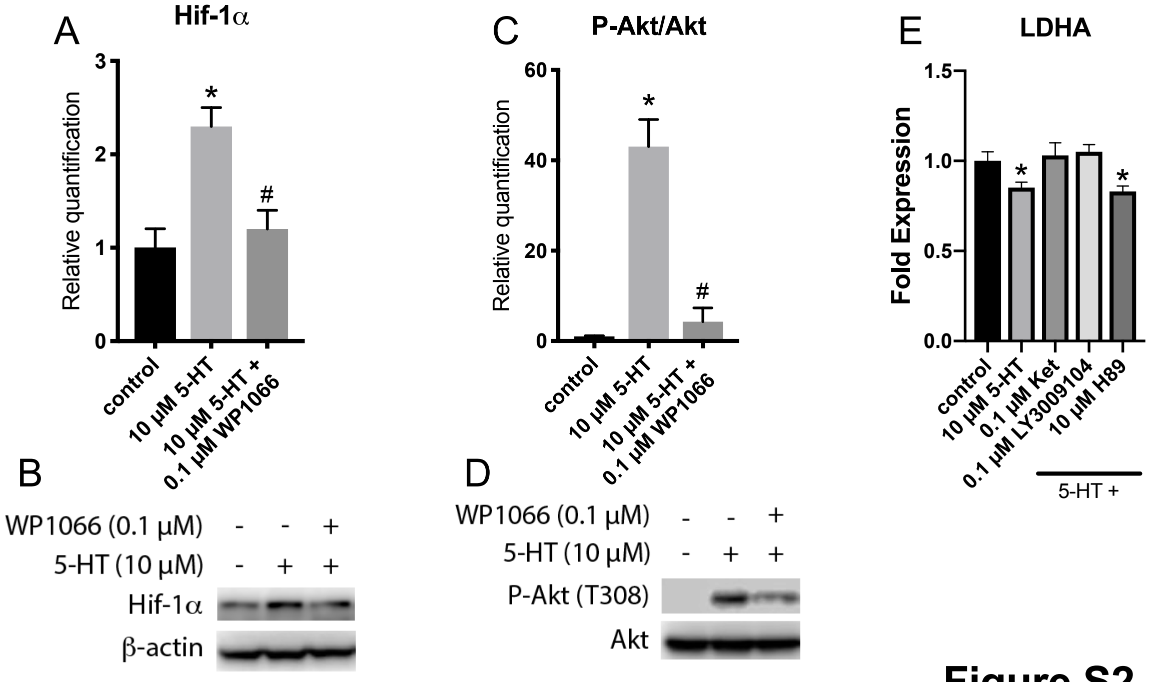

Figure S2

Fig. S2. Quantification (E) and representative Western blot (B) of Hif-1α expression. Quantification (C) and representative Western blot (D) of Akt phosphorylation. Plotted values represent means ± S.E.M. of 7 independent experiments (n=7). * and # P<0.05 as compared to control or to 10 μM 5-HT, respectively (One-way ANOVA followed by Tukey's post-test).(E) LDHA mRNA levels evaluated by qPCR. Plotted values represent means ± S.E.M. of 7 independent experiments (n=7; * P<0.05 as compared to control; one-way ANOVA followed by Tukey's post-test).
